# Molecular Docking Analysis of Pyrimethamine Derivatives with Plasmodium falciparum Dihydrofolate Reductase

**DOI:** 10.6026/97320630014232

**Published:** 2018-05-31

**Authors:** Indra Vikram Singh, Sanjay Mishra

**Affiliations:** 1 Laboratory of Bioinformatics, Department of Biotechnology, IFTM University, Delhi Road (NH 24), Moradabad 244 102, Uttar Pradesh, India

**Keywords:** Analogues, Antifolate, Antimalarial, de novo, DHFR, inhibitors, Plasmodium falciparum, resistance, quadruple mutant, validated targets

## Abstract

DHFR from Pf is a known target for malaria. There is a continued effort for the design and development of the potent inhibitor for PfDHFR in the control of malaria. Therefore it is of interest to screen PfDHFR with the derivatives of Pyrimethamine. The results show that the compound CID 10476801 has lowest docked energy (-11.48 kcal/mol) with protein likely to be a drug candidate, probably inhibiting PfDHFR structure. Residues of PfDHFR protein involved in the formation of hydrogen bonds with compound CID 10476801 are confirmed to be ASP54. The findings provide new insights into development of potent chemotherapeutic drug for combating malaria.

## Background

Malaria is an acute disease caused by mosquito. It has been a major global health problem of humans through history and is a leading cause of death across many tropical and subtropical countries. Over the last fifteen years renewed efforts made to control malaria have reduced the prevalence of malaria by over half, but the still its persistence, severity as well as emergence of resistance to existing drugs, there is a need to develop new drugs to combat this life threatening disease [1].

According to malaria world report 2016, it was estimated that 429 000 deaths from malaria occurred globally, a decrease of 50% since 2000 and of 22% since 2010. Most deaths in 2015 were estimated to have occurred in the WHO African Region (92%), followed by the WHO South-East Asia Region (6%) and the WHO Eastern Mediterranean Region (2%). Almost all deaths (99%) resulted from P. falciparum malaria. Plasmodium vivax is estimated to have been responsible for 3100 deaths in 2015, with most (86%) occurring outside Africa.

Malaria is caused by infection with protozoan parasites belonging to the genus Plasmodium transmitted by female Anopheles species of mosquitoes [2]. At present six plasmodia species including Plasmodium falciparum; Plasmodium vivax; Plasmodium ovale curtisi; Plasmodium ovale wallikeri; Plasmodium malariae; Plasmodium knowlesi out of which Plasmodium falciparum is usually considered the most important in terms of deaths [3].

Many drugs have been developed against malaria with the most important being chloroquine and artemisinin. The commonly used classes of antimalarial compounds include the quinolines (chloroquine, quinine, mefloquine, amodiaquine, primaquine), the antifolates (pyrimethamine, proguanil and sulfadoxine), the artmisinin derivatives (artemisinin, artesunate, artemether, arteether) and hydroxynaphthaquinones (atovaquine) [4]. The most widely used antimalarial drugs belong to the folate antagonist class, though their role in malaria control is laden by rapid emergence of resistance under drug pressure [5].

Antifolate antimalarial drugs interfere with folate metabolism, a pathway essential to malaria parasite survival. The antifolate drugs inhibit dihydrofolate reductase (DHFR) (pyrimethamine, cycloguanil) or dihydropteroate synthase (DHPS) (sulfadoxine), the two key enzymes in de novo folate biosynthesis; inhibition of this metabolic pathway leads to the inhibition of the biosynthesis of pyrimidines, purines, and some amino acids.

Currently, there are effective drugs to treat and control malaria; However, the ability of P. falciparum in particular to develop resistance to these treatments has threatened their continuing efficacy and raised the importance of combinations as well as developing new drugs and novel targets [6].

The Resistance to these drugs has arises rapidly and is now common worldwide. Resistance is caused by point mutations in DHFR and DHPS, the two key enzymes in the folate biosynthetic pathway that are targeted by antifolates [4]. Resistance to DHFR and DHPS inhibitors is conferred by single mutations of the gene encoding for the respective enzyme, resulting in substitutions in the amino acid chain [7].

New antimalarial treatments should display novel mechanisms of action with efficacy against already existing multi-drug resistant strains. Additionally, the interruption of parasite transmission, with the potential to contribute to malaria eradication, should be exploited by the next generation of antimalarial drugs [8].

The identification of new target for anti-malarial drugs for malaria elimination requires an integrated strategy, including new and old drugs, vaccines, vector control and public health measures. Considering the high mortality, morbidity, the emergence and spread of resistance to existing drugs, there is no question that new drugs are required [9]. To achieve this goal, anti-malarial drug research should focus on validated targets in order to generate new drug candidates [9].

## Methodology

### Receptor x-ray structure

The 3D coordinates of the crystal structure of Quadruple mutant (N51I+C59R+S108N+I164L) Plasmodium falciparum dihydrofolate reductase- thymidylate synthase (PfDHFR-TS) complexed with WR99210, NADPH, and dUMP (PDB id: 1J3K) was retrieved from PDB (http://www.rcsb.org) and taken as the receptor model in flexible docking program.

### Active site analysis

The active site residues of Quadruple mutant (N51I+C59R+S108N+I164L) Plasmodium falciparum dihydrofolate reductase-thymidylate synthase (PfDHFR-TS) was taken from the PDBSUM entry of 1J3K having binding site residues ASP54, CYS15, ILE14, LEU164, ASN108, PHE58, PRO113, ILE112 and MET55 for inhibitor WRA (6,6-dimethyl- 1- [3-(2,4,5-trichlorophenoxy) propoxy]-1,6-dihydro- 1,3,5- triazine-2, 4-diamine).

### Inhibitors Dataset

Twenty-six analogues of Pyrimethamine with experimentally derived quadruple mutant PfDHFR pKi values were obtained from the literature [10]. The 3D structures of known inhibitors were downloaded in .sdf format from pubchem compound database. They were later converted in .pdb format by the help of open babel [11] software.

### Molecular docking

Docking of twenty six analogues of Pyrimethamine screened from literature against Plasmodium falciparum DHFR structure was done using molecular docking program AutoDock [12]. Gasteiger charges are added to the ligand and maximum 6 numbers of active torsions are given to the lead compounds using AutoDock tool (http://autodock.scripps.edu/resources/adt). Kollman charges and the solvation term were then added to the protein structure using the same. We have made the grid and adjusted the number of points in X, Y, Z-axis so that the entire active site residue (ASP54, CYS15, ILE14, LEU164, ASN108, PHE58, PRO113, ILE112 and MET55) of the DHFR is covered. The Lamarckian genetic algorithm implemented in Autodock was used. Docking parameters were as follows: 30 docking trials, population size of 150, maximum number of energy evaluation ranges of 25,0000, maximum number of generations is 27,000, mutation rate of 0.02, cross-over rate of 0.8, Other docking parameters were set to the software's default values. After docking, the ligands were ranked according to their docked energy as implemented in the AutoDock program. Further the best-docked complexes were analyzed through Python Molecular Viewer [13] software for their interaction studies.

## Results & Discussion

**Figure 2:**
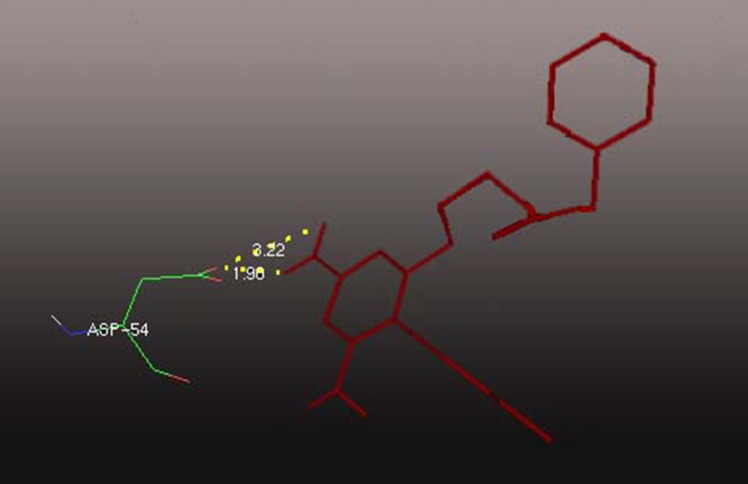
Docked complex showing the interaction between PfDHFR and the inhibitor compound (CID 10476801). Dotted yellow lines indicate the hydrogen bonding between amino acid residue ASP54 and the compound CID: 10476801.

Plasmodium parasites have an unmatched track record of gaining resistance to virtually all available drugs developed against them [14]. Over time, these parasites have acquired intricate strategies through which they continue to exercise their stubborn nature as colonists of their hosts [15, 16]. In the present investigation, docking experimentation revealed the interaction of ligands with protein and residues involved in this complex. For such interaction studies, the most important requirement was the proper orientation and conformation of ligand, which fitted to the enzyme binding site appropriately and formed protein-ligand complex. Therefore, optimal interactions and the best autodock score were used as criteria to interpret the best conformation among the 30 conformations, generated by AutoDock program. The docking results of twenty-six compounds and one known inhibitor Pyrimethamine with PfDHFR were shown in Table 1. Among the above docked compounds CID 10476801 had the lowest docking energy with PfDHFR than other docked compounds. Therefore it was predicted that compound CID 10476801 has lowest docked energy (-11.48 kcal/mol) with protein was a drug candidate, which inhibit PfDHFR structure. Docking pose of the best conformation of compound CID 10476801 in the binding site of PfDHFR protein is shown in Figure 2. Taken together the present study with [17], some chemical modifications to address the identified undesirable properties may be necessary. Interestingly though, the hits had a cholesterol-like nucleus, and might be well tolerated by human subject to further investigation. Overall, as these compounds showed encouraging selectivity between human and plasmodial inhibitors like cysteine proteases Further, residues of PfDHFR protein involved in the formation of hydrogen bonds with compound CID 10476801 is ASP54. Hydrogen bonding plays an important role for the structure and function of biological molecules, especially for inhibition in a complex.

## Conclusion

The Plasmodium falciparum dihydrofolate reductase is a drug target for malaria. Docking study predicted that compound CID 10476801 has lowest docked energy with PfDHFR and the interaction is stabilized by hydrogen bonding. The findings provide new insights into development of potent chemotherapeutic drug for combating malaria.

## Figures and Tables

**Table 1:** The docking results of the twenty seven compounds with PfDHFR.

Sl. No.	CID No.	Binding Energy (Kcal/mol)	Intermol Energy (Kcal/mol)	Torsional Energy (Kcal/mol)	Internal Energy (Kcal/mol)	Docking Energy (Kcal/mol)
1	25099455	-8.84	-9.15	0.31	0.2	-8.96
2	23423608	-7.38	-8.32	0.93	-0.31	-8.63
3	10090457	-9.5	-11.05	1.56	-0.07	-11.12
4	9974420	-9.28	-10.52	1.25	-0.71	-11.23
5	10018953	-7.5	-9.37	1.87	-0.17	-9.54
6	23423607	-8.71	-9.33	0.62	-0.2	-9.52
7	11463215	-8.59	-10.46	1.87	-0.65	-11.11
8	10476801	-9.15	-11.64	2.49	0.17	-11.48
9	9814965	-9.47	-10.71	1.25	-0.42	-11.13
10	10902094	-8.78	-9.4	0.62	-0.22	-9.62
11	11152240	-8.75	-10.62	1.87	-0.44	-11.06
12	13926968	-8.15	-8.15	0	-0.08	-8.23
13	10266000	-7.05	-8.92	1.87	-0.37	-9.3
14	11369668	-7.47	-9.03	1.56	-0.14	-9.17
15	11290186	-6.81	-8.36	1.56	-0.02	-8.38
16	93114	-7.65	-7.96	0.31	-0.11	-8.07
17	29142	-8.53	-8.84	0.31	-0.09	-8.93
18	11369471	-7.61	-8.55	0.93	-0.48	-9.02
19	11121319	-9.54	-10.78	1.25	-0.55	-11.33
20	11020649	-7.69	-8	0.31	-0.1	-8.1
21	134626	-8.61	-8.92	0.31	-0.02	-8.94
22	11369471	-9	-9.94	0.93	-0.19	-10.13
23	10426185	-7.42	-9.29	1.87	-0.29	-9.57
24	13926986	-8.21	-8.83	0.62	0.15	-8.68
25	10927461	-6.95	-9.75	2.8	0.29	-9.46
26	10060600	-8.56	-8.87	0.31	-0.09	-8.96
27	4993	-8.3	-8.61	0.31	-0.07	-8.68

**Figure 1:**
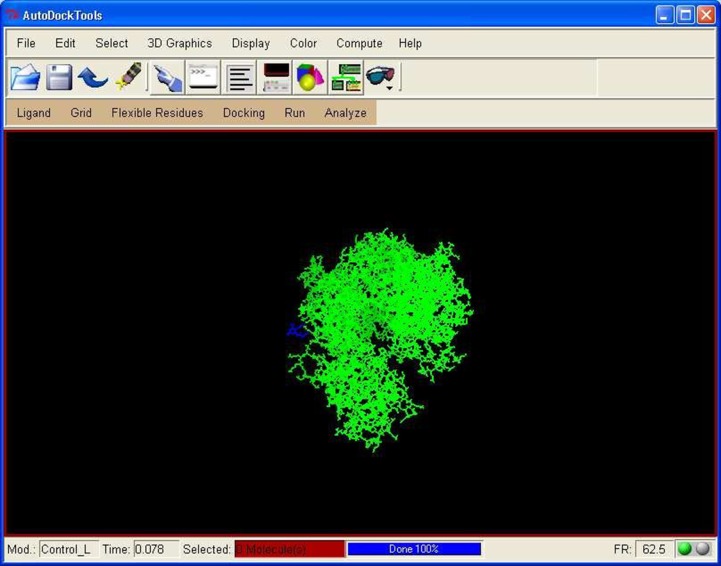
Molecular Interaction of ligand with native enzyme protein PfDHFR using Autodock.
